# Supplementary material for: Comprehensive analysis of chromosomal mobile genetic elements in the gut microbiome reveals phylum-level niche-adaptive gene pools
Source: PLoS One. 2019 Dec 12;14(12):e0223680. doi: 10.1371/journal.pone.0223680 (PMC6907783; doi:10.1371/journal.pone.0223680)
Supplement: S1 File — Figure A. Classification of gut microbiome MGEs at the phylum levelFigure B. Comparative genomics and alignment visualization to inspect MGE boundaries (DOCX) [file pone.0223680.s001.docx]

**
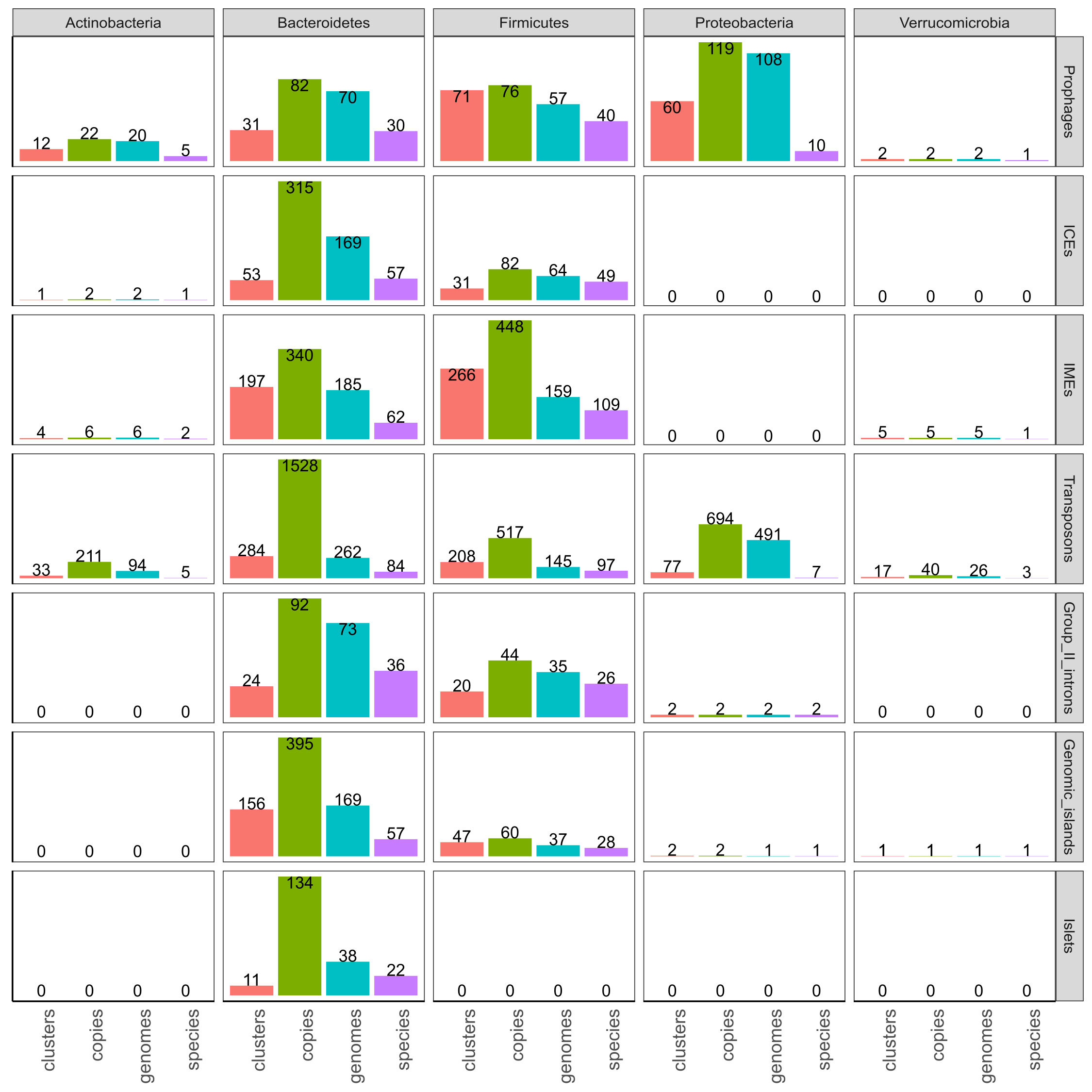
**

**Figures A. Classification of gut microbiome MGEs at phylum level.** The number of MGEs identified stratified by five phyla and MGE classification. The counts are measured at four levels: clusters, copies, genomes and species.

**
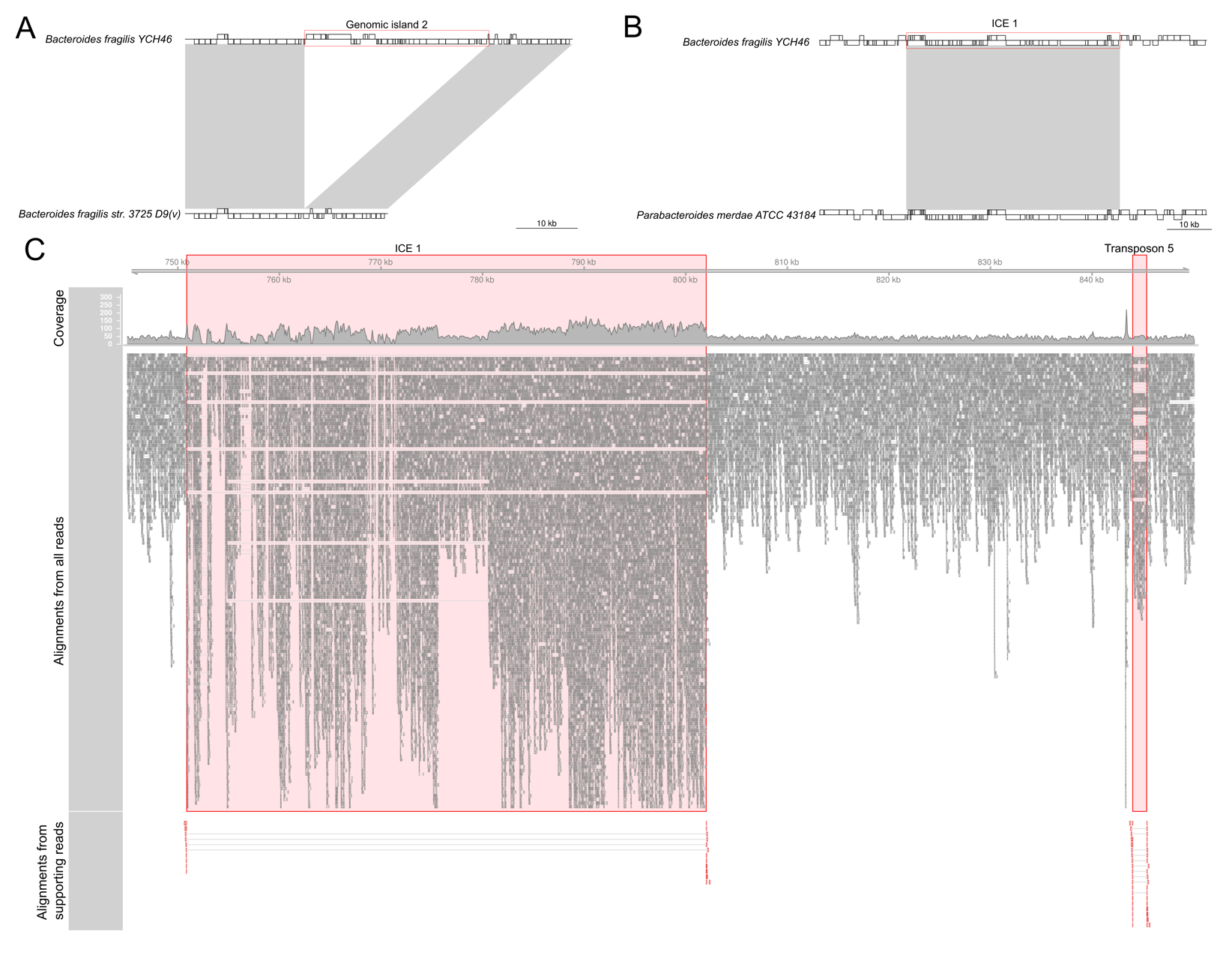
Figure B. Comparative genomics and alignment visualization to inspect MGE boundaries.** A) MGE insertion boundaries verified by synteny breaks in genomes of the same species. B) MGE boundaries verified by observing identical sequences in two different species. C) Multiple spit reads and discordant paired-end reads supporting insertion boundaries of MGEs into the reference genomes. Top panel: the coverages from all reads aligned to the reference genomes; middle panel: the alignments of all reads; bottom panels: the alignments from reads supporting the insertion of MGEs into the reference genome. Two inserted MGEs are highlighted in the red blocks in top two panels.
