# Supplementary material for: Comprehensive analysis of chromosomal mobile genetic elements in the gut microbiome reveals phylum-level niche-adaptive gene pools
Source: PLoS One. 2019 Dec 12;14(12):e0223680. doi: 10.1371/journal.pone.0223680 (PMC6907783; doi:10.1371/journal.pone.0223680)
Supplement: S1 Table — (DOCX) [file pone.0223680.s006.docx]

**S1Table. Antibiotic resistant genes identified in MGEs classes**

| MGE class | Total cargo genes # | Total ARGs # | Percentage (%) |
| --- | --- | --- | --- |
| Prophage | 14632 | 2 | 0.01 |
| ICE | 20269 | 140 | 0.69 |
| IME | 9302 | 66 | 0.71 |
| Genomic Island | 13083 | 34 | 0.26 |
| Islet | 208 | 1 | 0.48 |
| Transposon | 3860 | 9 | 0.23 |
